# A case report of breast cancer and membranous nephropathy with positive anti phospholipase A2 receptor antibodies

**DOI:** 10.1186/s12882-021-02511-x

**Published:** 2021-09-30

**Authors:** David Mathew, Sanjana Gupta, Neil Ashman

**Affiliations:** Department of Nephrology, Royal London Hospital, Whitechapel Road, London, E1 1FR UK

**Keywords:** Cyclophosphamide, Malignancy, Membranous, Nephrotic, PLA2R, Primary, Remission

## Abstract

**Background:**

Testing for antibodies against podocyte phospholipase A2 receptor-1 (PLA2R) allows clinicians to accurately identify primary membranous nephropathy (MN). Secondary MN is associated with a spectrum of pathology including solid organ malignancy. PLA2R positivity in these patients occurs, although no case of PLA2R-positive MN has been definitively linked to cancer.

**Case presentation:**

We describe a case of biopsy-proven PLA2R-positive MN, in whom invasive ductal carcinoma of the breast was discovered. The patient underwent surgery and adjuvant chemotherapy (including cyclophosphamide) and went into a sustained complete remission of her nephrotic syndrome.

**Discussion and conclusions:**

Case series have reported PLA2R positivity in patients with solid organ malignancy associated MN. Our case is unusual as it is a breast malignancy, and the patients nephrotic syndrome and anti-PLA2Rab titres improved with treatment of the cancer. Here we report, to the best of our knowledge, the first case of oestrogen receptor-2 positive breast cancer associated with PLA2R positive MN in a young lady that was treated successfully by treating the malignancy.

## Background

Antibodies against podocyte phospholipase A2 receptor-1 (PLA2R [1]) were discovered in 2009. Testing for PLA2R antibody allows clinicians to quickly and accurately (specificity approaching 100% [2]) identify primary membranous nephropathy (MN). Secondary MN is associated with a spectrum of pathology including solid organ malignancy. PLA2R positivity in these patients occurs, although no case of PLA2R-positive MN has been definitively linked to cancer [3]. We describe a case of biopsy-proven PLA2R-positive MN, in whom invasive ductal carcinoma of the breast was discovered. The patient underwent surgery and adjuvant chemotherapy (including cyclophosphamide) and went into a sustained complete remission of her nephrotic syndrome.

## Case report

A 42 year old Black British woman with no previous medical history of note presented with the nephrotic syndrome (albumin 28 g/L, urine protein creatinine ratio (uPCR) 650 mg/mmol and cholesterol 11.3 mmol/L). Excretory renal function was preserved with estimated glomerular filtration rate (eGFR) > 60 mL/min/1.73m^2^.

She described 2 months of leg swelling with no other associated symptoms; physical examination identified ankle oedema and hypertension with a blood pressure of 152/82 mmHg.

Further laboratory testing to investigate her nephrotic syndrome was as follows: Hepatitis B Surface Antigen negative, Hepatitis B Core Antibody negative, Hepatitis C Antibody and HIV serology negative. Anti-Nuclear Antibody negative, Extractable Nuclear Antigen negative, Double stranded DNA negative and Rheumatoid Factor undetectable. Immunoglobulin A 2.33 g/L, Immunoglobulin G 7.8 g/L, IgG Subclass 4 0.349 g/L, Immunoglobulin M 0.96 g/L, C3 1.46 g/L, C4 0.47 g/L. No light chains detected on serum or urine protein electrophoresis.

An anti-PLA2R antibody titre was measured at 178kunits/L by ELISA.

Renal biopsy demonstrated characteristic capillary loop thickening, spike formation on silver stain and positive immunohistochemistry for anti-PLA2Rab with polytypic IgG4. A diagnosis of primary MN was made.

Her blood pressure and volume overload were controlled on irbesartan and furosemide. Anticoagulation was declined by the patient even when her albumin dropped to < 25 g/L. The expected hypercholesterolaemia was managed with atorvastatin.

Despite maximal non-immunosuppressive anti-proteinuric treatment the patient’s nephrosis persisted, and worsened. Her serum albumin fell to 18 g/L, uPCR increased to 950 mg/mmol and anti-PLA2Rab rose on serial testing to 448kunits/L. Eleven months after her initial presentation, in this context, it was agreed with her to treat with immunosuppression. Initiation of this regime was delayed at the patients request. Two months after this decision had been made and, prior to the commencement of any immunosuppression therapy, the patient was diagnosed with multifocal grade 2 invasive ductal carcinoma of the right breast. This was estrogen receptor positive and human epidermal growth factor negative and staging revealed no metastatic disease (pT2 pN1 M0).

She underwent curative treatment with a right mastectomy and axillary lymph node clearance followed by chemotherapy and chest wall radiotherapy. Post-operatively and prior to adjuvant chemotherapy with intravenous cyclophosphamide and doxorubicin she remained nephrotic. She then completed 6 cycles of chemotherapy and received a total cyclophosphamide dose of 6.4 g with doxorubicin 0.64 g.

Clinical improvement of MN timed to successful treatment of the underlying malignancy. After completion of chemotherapy her serum albumin had increased to 34 g/L, the uPCR had improved to 512 mg/mmol (peak 1400 mg/mmol) and the anti-PLA2Rab titre fell to 4kunits/L (peak titre 674kunits/L). Now, 18 months after completing therapy, her anti-PLA2Rab titre is < 2, with a normal serum albumin and a reducing urine PCR of 344 mg/mmol. She is now in a sustained partial remission from her MN.

## Discussion and conclusions

Case series have reported PLA2R positivity in patients with solid organ malignancy associated MN. In one [3], only 3 of 10 patients were positive both for serum anti-PLA2RAb and histological IgG4. These patients had stomach, lung and larynx malignancies. Our case is unusual as it is a breast malignancy, and her nephrotic syndrome and anti-PLA2Rab titres improved with treatment of the cancer. Additionally, our patient is young whereas the mean age of malignancy associated MN is 66.

The cyclophosphamide dose used to treat the breast cancer was a lower dose than that used to successfully treat primary MN; however the contribution of this treatment to the resolution of her nephrosis cannot be completely excluded, indeed there are reports of partial remission of MN with Cyclophosphamide doses of less than 3 g [4]. Although less likely given her high PLA2R titre, a spontaneous remission of primary MN is also possible independent of the malignancy.

Here we report, to the best of our knowledge, the first case of oestrogen receptor-2 positive breast cancer associated with PLA2R positive MN in a young lady that was treated successfully by treating the malignancy. We caution clinicians that the exclusive use anti-PLA2Rab in determining a diagnosis of primary MN may not be appropriate.

Case series have demonstrated an association between THSD7A and malignancy in MN [5] and the advent of laser capture microdissection and mass spectrometry has led to the identification of NELL1 as a putative biomarker for malignancy associated MN in PLA2R negative patients [6]. It is likely that further targets will be identified in the field of MN in the coming years which will further elucidate the association between this disease and malignancy.

## Data Availability

Data referred to from previously published work is referenced in the body of the text.

## Abbreviations

eGFR: Estimated Glomerular Filtration Rate
PLA2R: Phospholipase A2 Receptor-1
MN: Membranous Nephropathy
uPCR: urine Protein Creatinine Ratio
NELL1: Nerve Epidermal Growth Factor Like 1
THSD7A: Thrombospondin type-1 domain containing 7A
